# Optimizing Time-Sensitive Traffic Scheduling in Low-Earth-Orbit Satellite Networks

**DOI:** 10.3390/s25144327

**Published:** 2025-07-10

**Authors:** Wei Liu, Nan Xiao, Bo Liu, Yuxian Zhang, Taoyong Li

**Affiliations:** School of Information and Navigation, Air Force Engineering University, Xi’an 710003, China; lw1220359717@163.com (W.L.);

**Keywords:** LEO satellite network, time-sensitive network, software-defined network, queue scheduling

## Abstract

In contrast to terrestrial networks, the rapid movement of low-earth-orbit (LEO) satellites causes frequent changes in the topology of intersatellite links (ISLs), resulting in dynamic shifts in transmission paths and fluctuations in multi-hop latency. Moreover, limited onboard resources such as buffer capacity and bandwidth competition contribute to the instability of these links. As a result, providing reliable quality of service (QoS) for time-sensitive flows (TSFs) in LEO satellite networks becomes a challenging task. Traditional terrestrial time-sensitive networking methods, which depend on fixed paths and static priority scheduling, are ill-equipped to handle the dynamic nature and resource constraints typical of satellite environments. This often leads to congestion, packet loss, and excessive latency, especially for high-priority TSFs. This study addresses the primary challenges faced by time-sensitive satellite networks and introduces a management framework based on software-defined networking (SDN) tailored for LEO satellites. An advanced queue management and scheduling system, influenced by terrestrial time-sensitive networking approaches, is developed. By incorporating differentiated forwarding strategies and priority-based classification, the proposed method improves the efficiency of transmitting time-sensitive traffic at multiple levels. To assess the scheme’s performance, simulations under various workloads are conducted, and the results reveal that it significantly boosts network throughput, reduces packet loss, and maintains low latency, thus optimizing the performance of time-sensitive traffic in LEO satellite networks.

## 1. Introduction

The low-earth-orbit (LEO) satellite network, with its characteristics of low latency, high capacity, and seamless global coverage, has developed rapidly in recent years and has become a key technological means for achieving global wireless access [1,2]. With the widespread adoption of time-sensitive applications such as industrial IoT and remote control, users have imposed stricter requirements on the transmission capabilities of LEO networks for time-sensitive services [3,4]. However, the high-speed movement of satellites and frequent orbital changes lead to dynamic topology switching of inter-satellite links (ISLs) and satellite–terrestrial links (STLs), causing significant fluctuations in latency for multi-hop transmission paths [5]. Meanwhile, the limited on-board computing capabilities and the competition for uplink and downlink bandwidth further exacerbate the complexity of resource allocation [6]. Against this backdrop, how to achieve efficient scheduling of time-sensitive flows (TSFs) and ensure quality of service (QoS) has become a critical challenge that urgently needs to be addressed.

Traditional terrestrial time-sensitive network technologies (e.g., cyclic queue and forwarding, CQF, based on Ethernet [7]) rely on fixed paths and static priority scheduling to provide deterministic latency guarantees for different services. However, the highly dynamic nature and resource constraints of LEO satellite networks fundamentally undermine the applicability of CQF. Firstly, frequent satellite link switching leads to the failure of preset routing, and static queue scheduling cannot adapt to dynamic path changes, resulting in excessive transmission latency [8]. Secondly, the limited buffer capacity of on-board switches and unpredictable queue delays (e.g., buffer overflow caused by burst traffic) significantly impair deterministic forwarding capabilities [9]. Therefore, directly applying traditional terrestrial time-sensitive network technologies to LEO satellite networks faces adaptability challenges. It is necessary to develop new scheduling algorithms that account for the dynamic topology and resource constraints of satellite networks to improve reliability, reduce packet loss rates, and enhance timeliness.

Current research on resource scheduling for LEO satellite networks has made significant progress, with researchers significantly enhancing network transmission efficiency and QoS through innovative routing optimization and resource allocation strategies. For instance, in the context of STLs, Di et al. [10] proposed an integrated architecture for ultra-dense LEO networks and terrestrial networks, reducing end-to-end transmission latency through dynamic traffic scheduling. To address burst congestion in ISLs, a routing scheme [11] was developed that partitions the network into segments and directs traffic to low-load regions, effectively balancing link loads. Additionally, Sun et al. [12] combined routing with a joint wavelength allocation mechanism for multi-wavelength beam ISLs, achieving maximum resource utilization while meeting stringent latency constraints. For end-to-end scenarios, the eTEG maximum flow routing algorithm [13], based on a time-expanded graph model, was proposed to optimize traffic transmission and energy efficiency. To meet the latency-sensitive requirements of diverse services, Huang et al. [14] developed an AI-based multipath traffic scheduling method, modeling the problem as a Markov decision process to derive intelligent scheduling strategies. Similarly, Tao et al. [15] propose a software-defined networking (SDN)-based architecture that employs deep reinforcement learning to address load-balancing challenges. These studies have improved network resource utilization but lack guarantees for high-priority services.

For single-satellite performance optimization, Marcano et al. [16] proposed an optimization model to analyze queuing delay performance under limited buffer conditions, determining appropriate buffer sizes. Zhou et al. [17] addressed the resource constraints of satellite networks by designing a two-stage dynamic programming framework to achieve optimal data scheduling solutions. Additionally, Yan et al. [18] introduced an injection time planning mechanism to enhance network throughput by optimizing injection scheduling. In terms of latency optimization, Wang et al. [19] proposed a latency-optimal scheduling algorithm that approaches the global optimal solution within a limited number of iterations. Vasisht et al. [20] developed the L2D2 mechanism, leveraging geographically distributed ground stations to reduce downlink latency. However, these studies did not account for time-sensitive services with varying latency requirements. Therefore, further research is needed to explore differentiated traffic management for diverse TSFs.

To address the high-priority service guarantees and differentiated forwarding requirements for multi-level TSFs, existing studies have explored time-sensitive networking (TSN) and traditional Ethernet scheduling mechanisms. However, current algorithms have not fully considered the constraints of on-orbit resources or adequately leveraged the multipath and redundancy advantages of satellite networks. Moreover, there are still deficiencies in meeting the differentiated forwarding needs of multi-level TSFs, and scheduling mechanisms require further improvements to enhance traffic forwarding efficiency. To this end, this paper proposes a triad-priority cycle queue forwarding (TPC-CQF) mechanism, an enhancement of the CQF framework, which optimizes the scheduling capabilities for multi-level time-sensitive services through priority differentiation. This approach reduces packet loss rates while improving the throughput of LEO satellite networks without significantly increasing average latency.

The main contributions of this paper are as follows:

(1) Designed a management framework for LEO satellite networks tailored to time-sensitive services, incorporating SDN-based management strategies.
(2) Developed an efficient dynamic priority queue scheduling mechanism (TPC-CQF) to provide differentiated forwarding guarantees for multi-level time-sensitive flows.
(3) Proposed a flexible scheduling strategy based on redundant time slots, fully utilizing the redundant time slots of time-sensitive flows to achieve efficient forwarding of high-priority TSFs.

## 2. System Model

In this section, we first introduce the satellite–terrestrial network architecture and then discuss the queuing and forwarding mechanism for data flows.

### 2.1. Network Architecture

**Figure 1:**
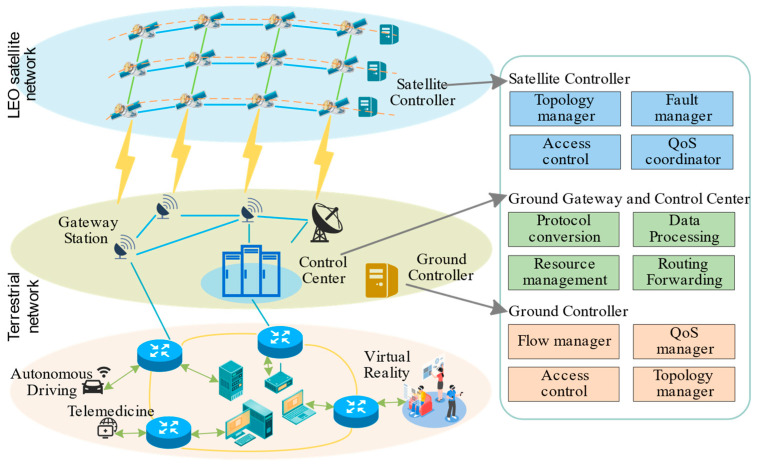
Satellite–ground network architecture model.

LEO satellite networks primarily consist of ground terminal users, LEO satellites, and ground control systems, as shown in Figure 1. To efficiently forward TSFs, this paper adopts a SDN-based architecture, decoupling the traditional network into a control plane and a data plane. The data plane handles data forwarding. Its core functions include inter-satellite data forwarding, load balancing, and data buffering, utilizing wireless and optical links to ensure efficient interconnection and low-latency communication between terminals and satellites while effectively coping with network dynamics. The control plane focuses on global network resource management, traffic scheduling optimization, and network topology adjustment.

The control plane consists of a globally distributed cluster of ground controllers located at gateway stations and lightweight satellite controllers (acting as local agents) deployed on each satellite. The ground controller cluster serves as the global network controller. Its primary responsibilities include managing terminal users. To achieve this, it interfaces with local network providers (LNPs) to obtain essential user context information, such as user location, access point identifiers, subscribed service level agreements (SLAs), and specific QoS requirements for TSFs. Simultaneously, the ground controller cluster leverages precise satellite orbit prediction models and periodically receives local state information (including topology segments, link load, queue status, etc.) reported by satellite controllers via STLs. It performs global optimization calculations to determine end-to-end paths and scheduling strategies. The resulting control information (e.g., routing policies and resource allocation schemes) is proactively distributed to the corresponding satellite controller via STLs during the satellite’s flyover of a ground station communication window. This design avoids the additional latency, reliability risks, and bandwidth consumption associated with relaying control signaling through ISLs. The satellite controller’s main tasks are to locally execute received control strategies, monitor real-time onboard status (including link state and load), manage ISLs and STLs, and perform data forwarding. Notably, when communication with the ground controller is interrupted or experiences high latency, the satellite controller can make localized decisions based on predefined policies, such as switching to backup paths or implementing local congestion control.

The data plane comprises onboard satellite switches and ground access equipment. Its core function is to perform real-time packet forwarding, load balancing, and buffer management locally using wireless and optical links, dynamically adapting to network changes and ensuring low-latency communication. The central research focus of this paper is the queue scheduling mechanism implemented within the data plane, aiming to guarantee the efficient forwarding of TSFs. The control plane issues routing policies, resource allocation schemes, and priority parameters to data plane components via SDN protocols (primarily transmitted over STLs). This collaborative operation between the data plane and control plane ensures the effective utilization of network capacity and traffic stability, thereby efficiently supporting the forwarding of diverse traffic flows, including TSFs.

### 2.2. Queue Scheduling Mechanism for TSFs

The topology of inter-satellite links (ISLs) in LEO networks is dynamic, influenced by satellite orbital motion, line-of-sight visibility, and relative positional changes. Consequently, routing strategies must adapt dynamically to real-time satellite position updates for efficient data transmission. This paper focuses on optimizing the node-level forwarding mechanism for delay-sensitive flows. Therefore, we employ a shortest-path routing algorithm. The SDN controller dynamically computes the optimal path, selecting one or multiple satellites as relay nodes to minimize forwarding delay. Given the distance instability caused by ISL switching and our primary focus on queue scheduling optimization, we simplify the propagation delay component. The total network delay comprises transmission delay and queuing delay, expressed as:

(1) $$D_{total}\;=D_{trans}\;+D_{queue}\;$$

Queue allocation management is divided into two main phases: packet enqueuing and packet dequeuing. Data flows in the queue can be broadly categorized into TSFs and non-sensitive flows (NSFs). The enqueuing phase involves selecting the target queue for newly arrived packets, taking into account the total priority sum of available queues, queue capacity, and packet collision issues. The dequeuing phase determines the queue from which packets are sent in each time slot, addressing cases where the total priority sums are equal and dynamically adjusting priorities. To ensure the prioritized transmission of delay-sensitive flows, factors such as the urgency of the flows and scheduling strategies must be comprehensively considered during scheduling to reasonably adjust the priority and scheduling order of each flow. Therefore, the following key parameters need to be defined:

Queue priority sum: The priority sum of queue *Q*, denoted as *S_Q_*, is defined as the sum of the dynamic priorities of all waiting packets in the queue:

(2) $$S_{Q}=\sum_{p\in D_{Q}}\pi_{p}\;\;$$

where *D_Q_* represents the set of all waiting packets in queue *Q*, $\pi_{p}$ denotes the dynamic priority of packet *p*, and the packet size is $s_{p}$; if the queue is empty, $S_{Q}=0\;\;$. When a new packet *p*1 arrives with a priority of $\pi_{p1}$, the queue priority sum is updated as follows:

(3) $${S_{Q}}^{’}=S_{Q}\;\;+\pi_{p1}$$

Queue capacity: The remaining capacity of queue *Q*, denoted as *K_Q_*, is defined as:

(4) $$K_{Q}=K_{\max,Q}-\sum_{p\in D_{Q}}s_{p}$$

where $K_{\max,Q}$ represents the maximum capacity of queue *Q*.

Available queues: For each packet’s size, the availability of queues *F_Q_* is determined based on the remaining capacity *K_Q_* calculated above:

(5) $$F_{Q}=\left\{K_{Q}\;\geq {\;s}_{p}\right\}$$

## 3. TPC-CQF Improved from CQF

In this section, we analyze the limitations of the CQF mechanism in existing time-sensitive networks and propose a dynamic priority queue scheduling mechanism aimed at providing differentiated forwarding for time-sensitive flows and leveraging delay-redundant time-sensitive flows to enhance overall scheduling capability.

### 3.1. CQF-Based Queue Scheduling Mechanism

**Figure 2:**
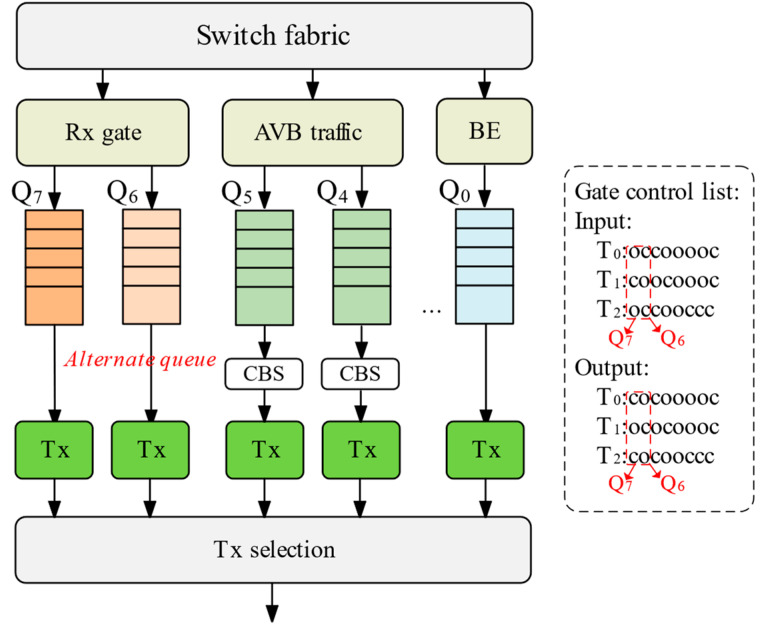
Traditional CQF queue scheduling mechanism.

CQF is a specific configuration of time-aware shaping (TAS), as shown in Figure 2.

The CQF mechanism distributes traffic across distinct queues, each handling specific traffic types. Queues Q_5_ and Q_4_ guarantee service for audio/video bridging (AVB) traffic, while Q_3_ reserves bandwidth for non-time-sensitive traffic. Queues Q_0_ and Q_2_ employ best-effort forwarding for regular Ethernet traffic, and Q_1_ handles the lowest-priority background traffic. Given the high latency sensitivity of time-sensitive flows (TSFs), they are assigned to high-priority queues (typically Q_6_ and Q_7_). These TSF queues operate under an XOR-gated mechanism: during fixed T-second time slots, one queue exclusively receives packets while the other transmits. This design ensures TSF forwarding within two slots. Assuming switch processing keeps pace, latency over *n* hops ranges between (n − 1)T and nT.

Despite guaranteeing TSF forwarding within bounded slots, the traditional CQF approach presents significant limitations. A key drawback is its inability to provide differentiated forwarding services for TSFs with varying priority levels. All TSFs are scheduled within the same queue pair (Q_6_/Q_7_), lacking the flexibility to meet diverse QoS demands effectively. Furthermore, under traditional scheduling, if multiple TSFs arrive simultaneously, subsequent packets may be discarded based solely on first-come first-served order. Consequently, leveraging TSF delay redundancy to reduce packet loss represents a key factor for enhancing overall scheduling capability. Addressing this trade-off—potentially sacrificing minimal end-to-end latency to improve TSF service reliability—constitutes a primary objective of this work.

### 3.2. TPC-CQF Mechanism

To address the limitations of the traditional CQF mechanism in satellite time-sensitive networks, we propose an improved algorithm based on CQF, termed TPC-CQF, aimed at optimizing the network throughput of TSFs in large-scale network scenarios. This approach seeks to enhance the processing capability of time-sensitive flows in satellite networks by introducing a differentiated forwarding mechanism and flexible scheduling strategies.

**Figure 3:**
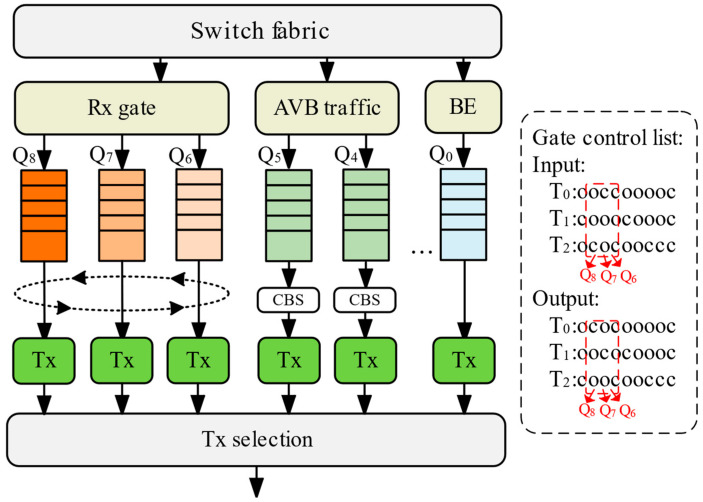
TPC-CQF scheduling mechanism.

The core improvement of our differentiated forwarding scheme enables priority-based handling for TSFs. As Figure 3 illustrates, a three-queue dynamic priority mechanism operates cyclically: during each time slot, one queue transmits packets while the other two receive incoming traffic (e.g., Q_1_ transmits; Q_2_ and Q_3_ receive). This architecture allocates packets to receiving queues and resolves contention conflicts. Key objectives include prioritizing high-priority TSF processing, preventing queue starvation, minimizing packet loss, and maximizing throughput.

Packet processing occurs in two phases: single-packet allocation and conflict resolution. New packets first verify remaining capacity K_Q_ in available queues, excluding those where $K_{Q}<s_{p}$ packet size. Allocation prioritizes the queue with the highest current priority sum S_Q_, denoted as $Q^{*}$. This strategy minimizes average waiting times for high-priority packets, as higher $Q^{*}$ values typically indicate either more urgent packets or longer-queued traffic. The mechanism simplifies tie-breaking and optimizes same-priority conflicts through packet size and queue suitability assessments.

(6) $$Q^{*}=\arg\underset{Q\in \{Q_{1},Q_{2},\ldots ,Q_{M}\}}{\max}S_{Q}$$

If multiple queues have the same S_Q_, assume the set of queues with equal S_Q_ is denoted as Set $F_{\mathrm{tie}}=\{Q_{i}|S_{Q_{i}}=S_{\max}\}$, where S_max_ is the highest priority sum value. Select the queue with the smallest remaining capacity K_Q_, denoted as Queue $Q^{*}$, to minimize wasted capacity.

(7) $$Q^{*}=\arg\underset{Q\in F_{tie}}{\min}K_{Q}$$

If the capacities are also equal, select the queue with the lowest occupancy rate (i.e., the queue with the fewest packets N_Q_) to balance the load and reduce queuing delay. The occupancy rate can be defined as the number of packets in the queue N_Q_. Assume the set of queues with equal S_Q_ and K_Q_ is denoted as Set $F_{\mathrm{tie}2}=\{Q_{i}|S_{Q_{i}}=S_{\max},K_{Q_{i}}=K_{\min}\}$, where K_min_ is the lowest remaining capacity value.

(8) $$Q^{*}=\arg\underset{Q\in F_{tie2}}{\min}N_{Q}$$

After each packet allocation or processing, update the queue’s priority sum and remaining capacity.

To handle conflicting packets (multiple packets arriving simultaneously), the following differentiated allocation rules are designed, categorized into two scenarios: packets with different priorities and packets with the same priority. For packets with different priorities, the allocation essentially follows the aforementioned principles, prioritizing high-priority packets. Therefore, additional consideration is needed for handling packets with the same priority. Assume that the dynamic priorities of packets p_1_ and p_2_ satisfy $\pi_{p_{1}}=\pi_{p_{2}}$. Then, evaluate $s_{p1}$ and $s_{p2}$, prioritizing the allocation of the larger packet to maximize queue utilization and enhance potential throughput. If $s_{p1}=s_{p2}$, randomly select one packet to enqueue first before processing the other.

For onboard queue buffer constraints in LEO satellite networks ($K_{Q}\leq K_{\max,Q}$), the fragmentation effect of satellite-borne buffers intensifies with increasing queue count *N*. Assuming TSF packet sizes follow a bimodal distribution, the overflow probability for bursty packets is:

(9) $$P_{overflow}=\exp\left[-\frac{{\left(K_{\max,Q}-\mu \right)}^{2}}{2\sigma^{2}}\right]$$

When N>3, $P_{overflow}$ exhibits exponential growth, violating the low packet loss rate design objective. Therefore, the three-queue architecture constitutes the optimal solution for balancing packet loss rate, latency, and complexity, a conclusion that will be validated in subsequent simulations.

**Figure 4:**
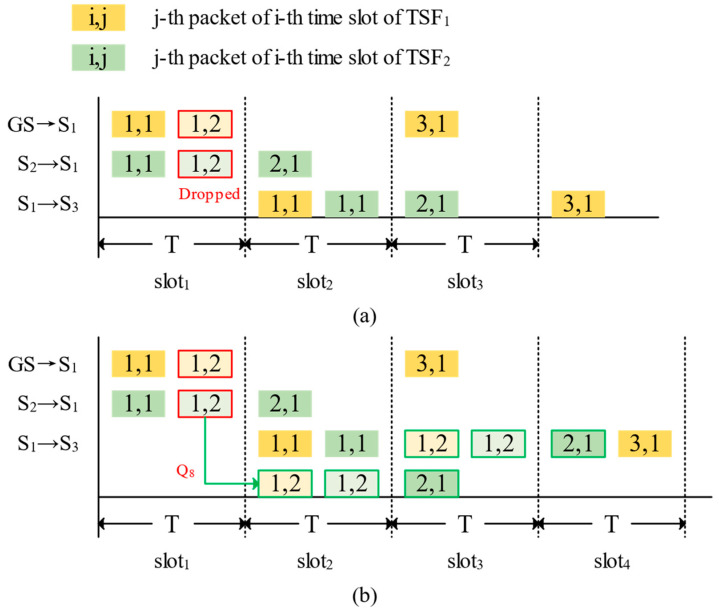
Packet scheduling of two time-sensitive flows: (**a**) CQF; (**b**) TPC-CQF.

Figure 4 illustrates that in the presence of packet collisions, TPC-CQF may slightly increase end-to-end delay but significantly improves resource utilization, enabling the transmission of more TSFs. For instance, in this scenario, two packets of TSF1 <1,2>, two packets of TSF2 <1,2>, and an additional packet <2,1> are all scheduled in Q8 for centralized forwarding in the subsequent time slot.

### 3.3. Queue Priority Control of TPC-CQF

The TPC-CQF control plane scheduling mechanism fundamentally differs from traditional CQF in time-sensitive traffic handling. Leveraging the SDN controller, TPC-CQF integrates path computation and flow scheduling management. This controller dynamically assigns flows to priority-tiered queues based on deadline-driven urgency. Furthermore, the queue priority design explicitly balances two critical considerations: packet loss risks from buffer constraints and mandatory service differentiation across priority levels.

In each time period, select queue $\arg\underset{Q\in \{Q_{1},Q_{2},\ldots ,Q_{M}\}}{\max}S_{Q}$ with the highest priority sum to transmit packets, optimizing the forwarding efficiency of high-priority packets. To prevent lower-priority packets from starving due to the continuous arrival of high-priority packets, a dynamic priority adjustment mechanism is introduced.

The number of waiting cycles N_P_ for packet P represents the number of time slots it waits:

(10) $$N_{p}=\left\lceil \frac{t_{\mathrm{current}}-t_{p,\mathrm{arrive}}}{T}\right\rceil$$

where T is the fixed time slot, $t_{p,\mathrm{arrive}}$ is the timestamp when the packet enters the queue, and $t_{\mathrm{current}}$ is the current time. Initial priority $\pi_{p}=\frac{1}{R_{p,\mathrm{remain}}}$ is adaptively promoted to ${\pi_{p}}^{’}$ according to job expiration thresholds and queuing duration, with synchronized recomputation of the global priority sum:

(11) $${\pi_{p}}^{’}=\frac{\pi_{p}}{1-N_{p}\cdot T\cdot \pi_{p}}$$

(12) $${S_{Q}}^{’}=\sum_{p\in D_{Q}}{\pi_{p}}^{’}\;\;$$

where $R_{p,\mathrm{remain}}$ denotes the initial packet deadline, which can be mapped to urgency levels via the SDN controller. Considering that ${\pi_{p}}^{’}$ may tend towards infinity, causing numerical instability, we impose a bounded constraint, $R_{p,\mathrm{remain}}-N_{p}\cdot T\geq \varepsilon$. Since queues forward packets within the next time slot at the earliest, the minimum setting for ε is 0.5. All aforementioned temporal parameters are measured in milliseconds (ms). Priority escalation of queues is verified and adjusted by the queue scheduling module.

Unlike TPC-CQF, the CQF mechanism exhibits inherent limitations for time-sensitive traffic. Simply increasing queue capacity cannot enhance forwarding performance because CQF mandates complete packet transmission within every time slot. Moreover, CQF cannot effectively prioritize flows with varying criticality levels. Crucially, it fails to leverage redundant time slots in TSFs, rigidly enforcing next-slot completion instead of flexible future slot utilization.

**Figure 5:**
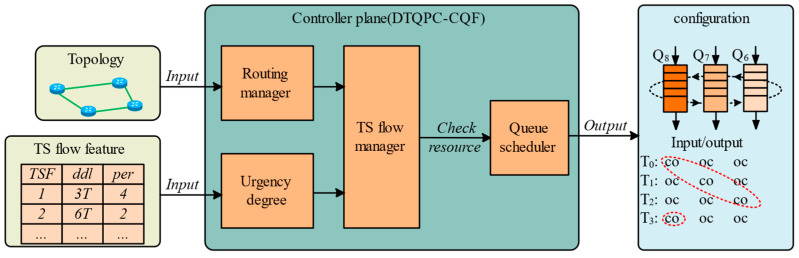
Queue priority control of TPC-CQF.

Figure 5 illustrates the queue priority control mechanism of TPC-CQF. Within each time slot, the state transitions of three queues are represented as ‘co’, ‘oc’, and ‘oc’. Here, ‘o’ indicates an open queue state, capable of receiving packets, while ‘c’ indicates a closed queue state, unable to receive packets. The two ‘oc’ states denote the queue’s open or closed status within the time slot, determining whether packets can be transmitted. Queues that are closed to receiving packets are assigned the highest priority. Once a queue is closed to receiving packets, the packets within that queue are guaranteed to be forwarded within the current time slot.

Regarding the aforementioned queue scheduling issue, as shown in Algorithm 1:

**Algorithm 1:** Queue Scheduling Algorithm Based on TPC-CQFInput: Packet set *P*, queue set *Q* = {*Q*1, *Q*2, *Q*3}, queue capacity $K_{\max,Q}$, current time slot *t*, fixed time slot duration T;
Output: Updated queue assignments, total priority sum SQ, remaining capacity KQ;
1:Initialize: For all Q = {Q1, Q2, Q3}, set S_Q_ = 0, KQ = $K_{\max,Q}$, Packet_Count [Q] = 0, Packet_Assignments [p] = Ø;2:for3:      For each packet p ∈ P arriving in time slot t: compute N_P_ and $\pi_{p}$ as well as FQ;4:      if $F_{Q}=\emptyset$ do5:            Discard p;6:      Else7:            Select $Q^{*}$ with the highest S_Q_ from F_Q_;8:            if SQ values are equal do9:                  Select $Q^{*}$ with the smallest K_Q_;10:                        if K_Q_ values are equal do11:                              Select min(PacketCount [Q]);12:      end if13:      Assign p to $Q^{*}$, update: K_Q_ [$Q^{*}$]− = $s_{p}$, S_Q_ [$Q^{*}$] += $\pi_{p}$, PacketCount [$Q^{*}$] += 1, PacketAssignments [p] = $Q^{*}$;14:      For each conflicting packet pair (p1, p2) ∈ P:15:      if $\pi_{p_{1}}>\pi_{p_{2}}$ do16:            Prioritize processing p1 before p2, go to step 2–12;17:      else if $\pi_{p_{1}}=\pi_{p_{2}}$ do18:            if $s_{p1}>s_{p2}$ do19:                  Prioritize processing p1 before p2, go to step 2–12;20:            else if $s_{p1}=s_{p2}$ do21:                  Randomly select one to process first, then the other;22:            end if23:      end if24:Select $\arg\max(S_{Q})$ for transmission25:endfor26:Update queue S_Q_, K_Q_;


Algorithm 1 dynamically selects the transmission queue based on the maximum or total priority of packets in each queue. The specific algorithm steps are as follows:

(1) Initialization: Set the total priority sum for all queues to 0, capacity to the maximum value, and packet count to zero. Initialize an empty packet assignment record.
(2) Calculate the waiting cycle based on the packet arrival time and the current time. Use the waiting cycle and deadline to compute the dynamic priority, determining the assignment order.
(3) Identify the set of feasible queues with sufficient capacity. From this set, sequentially select the queue with the highest total priority sum, the smallest capacity, and the fewest packets for packet assignment.
(4) Check the priority or size differences between packet pairs, prioritizing the processing of packets with higher priority or larger size. If both are equal, randomly select one for processing.
(5) Select the queue with the highest total priority sum to transmit its packets. After transmission, update the queue’s capacity, total priority sum, and packet count.
(6) Recalculate the waiting cycle and dynamic priority for packets in each queue. Update the queue’s total priority sum to reflect the latest state.

The time complexity of Algorithm 1 is *O*(*M*), where M denotes the maximum number of packets arriving in a single time slot. When packet collisions occur, the worst-case scenario requires $\frac{M(M-1)}{2}$ comparisons. Through priority pre-sorting optimization, the complexity is reduced to *O*(*MlogM*).

## 4. Simulation Analysis

### 4.1. System Design

In this section, we evaluate the performance of the proposed TPC-CQF algorithm through simulation and compare it with several typical TSN techniques. The evaluation primarily focuses on the following key metrics: packet timeout ratio, packet loss rate, end-to-end delay, and system throughput. Additionally, we analyze the algorithm’s scalability under varying load conditions. For comparison, we select the following baseline algorithms: CQF based on the IEEE 802.1 Qch standard [21] and traditional Ethernet switch (ES). The ES treats all traffic as regular traffic without distinguishing time-sensitive characteristics, processing it solely on a first-in–first-out (FIFO) basis. We compare the TPC-CQF algorithm with these two algorithms, focusing on the timeout ratio, packet loss rate, average delay, and throughput of the traffic. The simulation experiments are conducted on a personal laptop with an Intel Core i7-14650HX CPU and 32 GB of memory. The simulation environment is developed using PyCharm 2023.2.5 Community Edition, with core logic implemented in Python version 3.11.6, and integrates Systems Tool Kit (STK) software version 11.6 to generate the topology and dynamic characteristics of the satellite network.

In the simulation scenario, we designed a satellite network based on a Walker constellation for testing. Each satellite is equipped with four ISLs, connecting to the preceding and following satellites in the same orbit, as well as the left and right satellites in adjacent orbits. Some constellation parameters are shown in Table 1.

To simulate realistic satellite network traffic, we adopted an “ON/OFF” model to generate traffic flows. All compared techniques use the same random seed, ensuring that the traffic time series for all algorithms are identical. Packet sizes follow a bimodal distribution, while flow sizes follow a Pareto distribution. The ratio of TSFs to regular flows is 1:4. The network normalized load is controlled between 0.1 and 1, where the normalized load is defined as the ratio of the average generated traffic to the uplink bandwidth. We assume 100 users randomly generate traffic within the coverage area of each satellite. The deadlines for TSFs are randomly generated between 2 ms and 8 ms, excluding propagation delay, while the non-TS flow has an out-of-date latency of 1000 ms, which is the retransmission time-out latency of TCP. All flows are assumed to be unicast flows, with multicast flows monitored by decomposing them into multiple unicast flows.

### 4.2. Performance Results Analysis

We first validated the optimality of introducing three queues in TPC-CQF. Under a load of 0.8 and an ISL buffer size of 153.6 KB, it is observed that TPC-CQF achieves the optimal trade-off among packet loss rate, latency, and throughput. It is noteworthy that for the ISL buffer size, primarily selected as 153.6 KB to simulate payload-constrained satellite scenarios, this capacity satisfies moderate burst traffic requirements. A buffer size of 51.2 KB was employed to verify the algorithm’s robustness under more stringent resource constraints.

Table 2 clearly demonstrates the critical impact of queue count on TPC-CQF performance. While the dual-queue configuration achieves the lowest latency (1.82 ms), it exhibits excessively high TSF packet loss rate (14.8%) and timeout rate (7%). The five-queue configuration suffers from comprehensive performance degradation due to fragmentation (9.6% packet loss, 43% timeout rate, with throughput gain plummeting to +5%). The four-queue solution delivers intermediate performance (16% throughput gain, 2.1% packet loss) but remains significantly inferior to the three-queue design. This validates that the three-queue architecture represents the optimal balance between mitigating onboard buffer fragmentation effects (Equation (9)) and satisfying differentiated scheduling requirements for multi-level time-sensitive flows.

**Figure 6:**
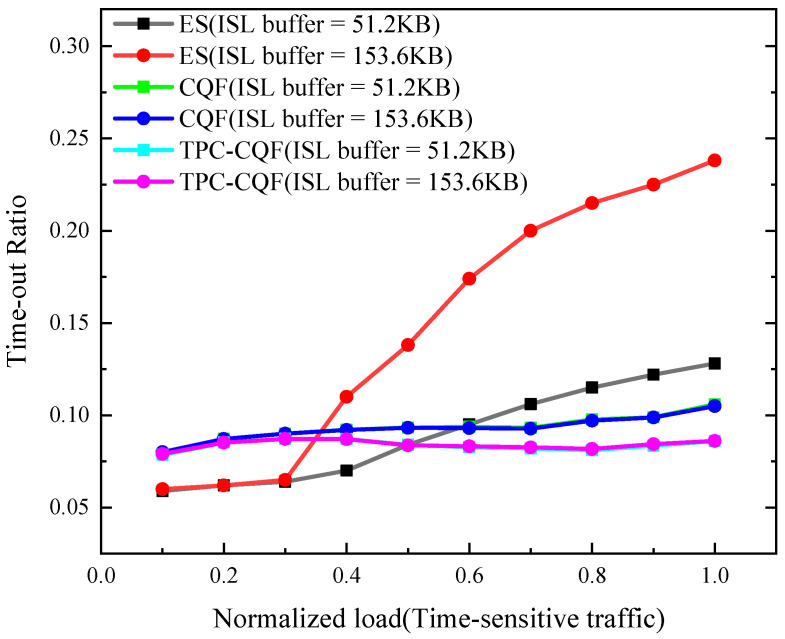
Timeout ratio.

To evaluate the forwarding capability for time-sensitive flows, we analyzed the service timeout ratio, defined as the proportion of packets that fail to reach the target node before their deadline. Figure 6 shows that as the load increases, the timeout ratio of TPC-CQF is significantly lower than that of traditional CQF and ES, and demonstrates robust performance under both 153.6 KB and 51.2 KB ISL buffer sizes. This is attributed to TPC-CQF’s introduction of a three-queue dynamic priority mechanism (Q_6_, Q_7_, Q_8_), which enables temporary storage of overloaded packets with delay tolerance and forwards them in the next time slot. This mechanism effectively utilizes the delay redundancy of time-sensitive flows (e.g., a deadline of 6T), significantly reducing timeout occurrences. Under the same buffer size, TPC-CQF’s timeout ratio is 10–18% lower than that of CQF, with a more pronounced advantage at high loads. The traditional Ethernet switch (ES) struggles to ensure timely packet transmission. As the load increases, the timeout ratio of ES exceeds 0.2.

The packet loss ratio is a key metric for evaluating network reliability, with packet losses occurring in both ISLs and STLs due to buffer overflow. We calculated two types of packet loss rates: one for the total traffic and one specifically for TSFs.

**Figure 7:**
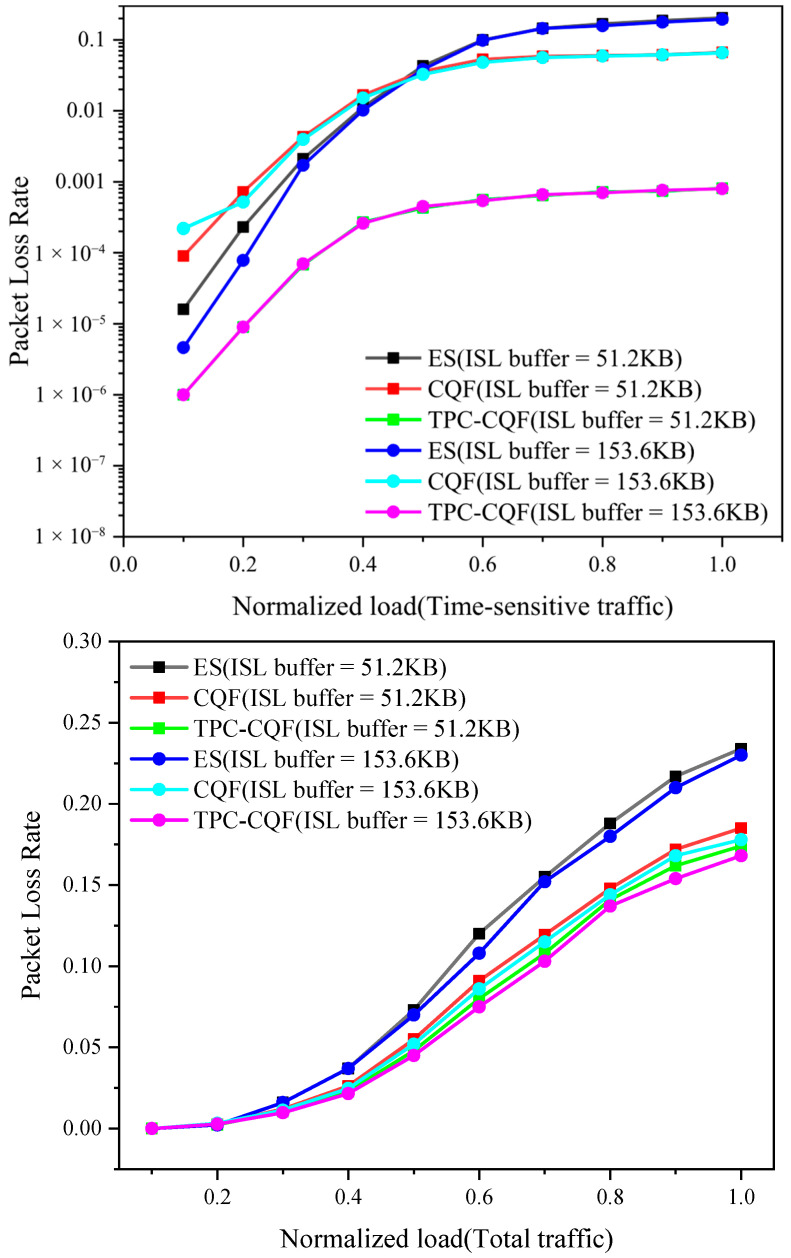
Packet loss ratio.

As shown in Figure 7, TPC-CQF exhibits a lower packet loss ratio for TSFs compared to CQF and ES across various loads, maintaining relatively stable performance under different ISL buffer sizes. Additionally, TPC-CQF demonstrates the best performance in terms of packet loss ratio for total traffic. While CQF can also reduce packet loss ratios, its performance for TSF packet loss is the worst under low load conditions due to its strict gating mechanism and limited queue forwarding capability for time-sensitive flows.

Additionally, we investigated packet loss performance under varying ISL buffer sizes. The core advantage of TPC-CQF lies in its differentiated forwarding and flexible scheduling. The traditional CQF’s dual-queue mechanism is prone to packet loss due to buffer overflow under high load or bursty traffic, whereas TPC-CQF’s three-queue design effectively mitigates this issue. The packet loss ratio for TSFs is below 0.001, representing a reduction of over 95% compared to CQF. The improvement in packet loss for total traffic is attributed to enhanced resource utilization. Although the introduction of the Q_8_ queue may slightly increase queuing delay for regular flows, the overall packet loss ratio remains comparable to that of CQF.

**Figure 8:**
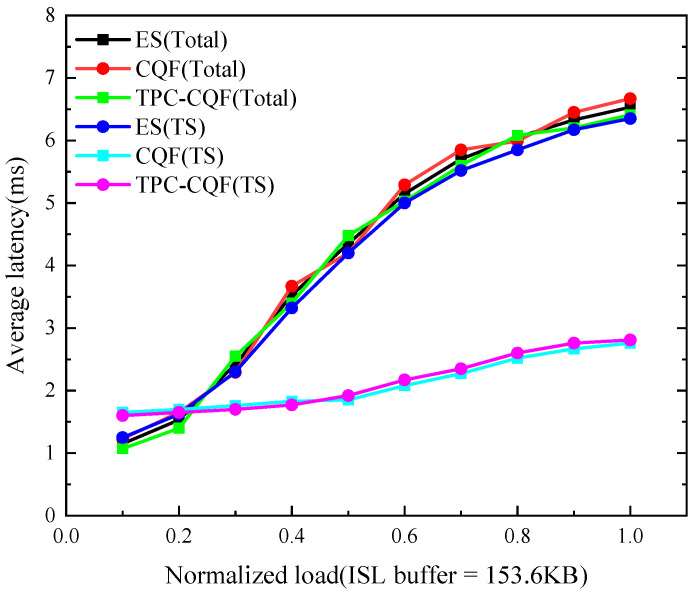
Average delay.

The average delay reflects the time from packet transmission to reception (excluding propagation delay). We calculated the average delay for both TSFs and total traffic across different mechanisms. Figure 8 shows that TPC-CQF’s TSFs delay is lower than that of ES but slightly higher than CQF under high load, while the delay for total traffic may be slightly higher.

The delay performance of TPC-CQF is directly related to its algorithmic design. Traditional CQF maintains lower TSFs delay through strict gating and dual-queue switching but sacrifices packet loss performance. In contrast, TPC-CQF achieves a lower packet loss ratio and forwards more traffic under the same load, resulting in a slightly higher average delay compared to CQF. TPC-CQF optimizes the packet loss rate by utilizing the Q_8_ backup queue, at the cost of delaying some packets by one time slot T (e.g., from slot 1 to slot 2). This results in TSF average delay being slightly higher than CQF under high load but still significantly lower than ES. The slightly higher delay for total traffic is due to the additional queue for handling TSFs, which increases the queuing time for regular traffic, thereby impacting the overall average delay.

**Figure 9:**
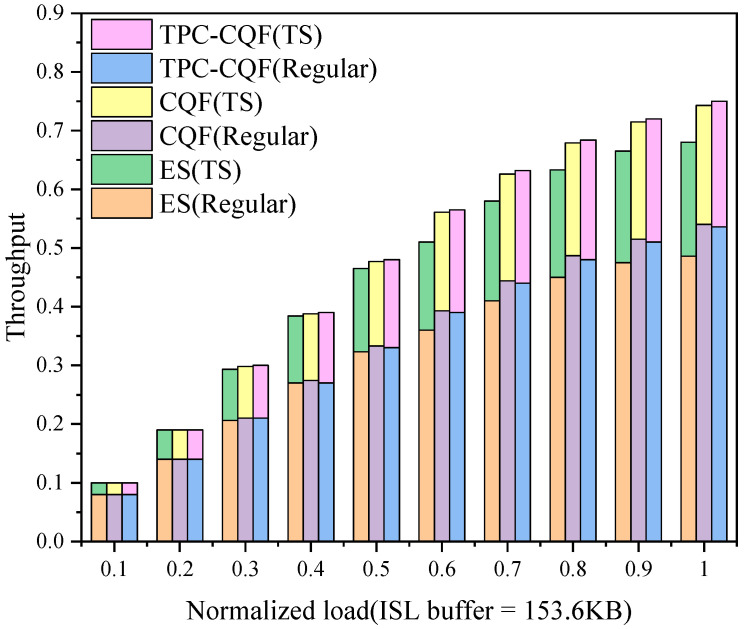
Throughput.

Throughput is a measure of the network’s transmission capacity, and we calculated it separately for TSFs and non-TSFs (regular traffic). Figure 9 shows that TPC-CQF’s TSF throughput significantly increases with load, and its overall throughput surpasses that of CQF.

The throughput improvement of TPC-CQF stems from its differentiated forwarding mechanism and flexible scheduling strategy. Traditional CQF is limited by packet loss under high load, which restricts TSF throughput. In contrast, TPC-CQF leverages the Q8 backup storage and dynamic priority scheduling to fully utilize network resources. The results demonstrate that TPC-CQF effectively enhances the scheduling capability for TSFs in large-scale satellite networks while maintaining high overall network performance.

Additionally, we evaluated the scalability of TPC-CQF across different constellation scales. Table 3 data shows that when satellite nodes increase from 64 to 225, the TSF packet loss rate rises from 0.02% to 0.36%, average latency increases by only 3.4%, and the timeout rate escalates from 7.0% to 13.6%. Although performance metrics exhibit slight fluctuations with scale expansion, the packet loss rate consistently remains below 0.4%, demonstrating the sustained effectiveness and stability of its queuing management mechanism in large-scale networks.

The results demonstrate that the TPC-CQF method delivers significant improvements in timeout ratio and packet loss ratio performance, addressing core limitations of conventional CQF in satellite time-sensitive networks (high packet loss, limited forwarding capacity). This enhancement stems fundamentally from TPC-CQF’s three-queue dynamic scheduling architecture and its integrated priority management, conflict resolution, and redundant slot utilization strategies, which effectively address the challenges of LEO satellite network link dynamics and onboard resource constraints.

## 5. Conclusions

This paper proposes an enhanced cyclic queue forwarding mechanism, TPC-CQF, to address the efficient forwarding and scheduling of TSFs in LEO satellite networks. By introducing a differentiated forwarding mechanism and dynamic priority queue scheduling, TPC-CQF effectively improves TSF transmission efficiency and quality of service. Simulation results show that compared to traditional CQF, TPC-CQF excels in reducing packet loss rates and enhancing throughput while having a minimal impact on average delay. Future research will focus on further optimizing the relationship between queue resources and network performance, with an emphasis on adaptive queue management strategies to enhance the algorithm’s robustness in mixed-traffic environments.

## Figures and Tables

**Table 1:** Constellation parameters.

Parameter	Value
Number of satellites	64
Number of orbits	8
Satellites per orbit	8
Orbit altitude	550 km
ISL bandwidth	1 Gbps
Uplink bandwidth per user	100 Mbps
Queue buffer capacity	32 KB
CQF time slot	500 microseconds

**Table 2:** Impact of queue count on scheduling performance.

Queue Count	TSF Packet Loss Rate	Average Latency (ms)	Throughput Gain	Timeout Rate
2	0.148	1.82	Baseline	7%
3	0.001	2.05	+28%	6.7%
4	0.021	2.91	+16%	12%
5	0.096	3.72	+5%	43%

**Table 3:** Performance metrics across constellation scales.

Satellite Node Count	TSF Packet Loss Rate	Average Latency (ms)	Throughput	Timeout Rate
64	0.0002	2.05	78.2%	7.0%
100	0.0008	2.08	67.8%	9.3%
225	0.0036	2.12	55.6%	13.6%

## Data Availability

The original contributions presented in the study are included in the article, further inquiries can be directed to the corresponding author.
